# 

**Published:** 1911-12

**Authors:** 


### OUR INDIANA CORRESPONDENT.

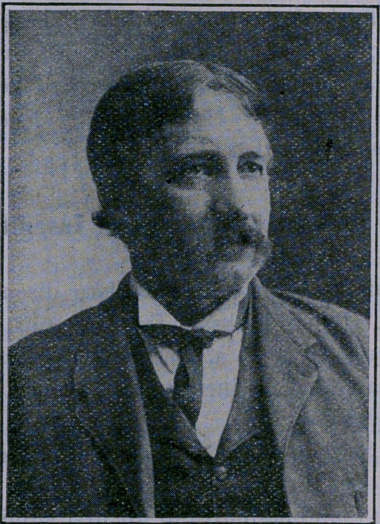

DR. G. HENRI BOGART, TERRE HAUTE, INDIANA,
Physician, Author, Poet and Humanitarian, Apostle of Socio-Sexual Reform, Hygiene and Eugenics.

Dr. G. Henri Bogart was born in Cincinnati, Ohio, of Holland Dutch and Scotch ancestry, and graduated from high school at 12 years of age. He studied medicine both as an eclectic and an allopath, and holds himself absolutely independent. He has been for many years a student of eugenics and sociology, and as such has been one of the earliest and most radical of the promoters of the "sterilization of the unfit" doctrine.

Dr. Bogart asserts that whenever he sees a degenerate, and then considers the multitude who never will be born through the operation of this law, that he would not exchange his world influence in this line for all the wealth and benefactions of Carnegie.

His entrance into the field of general eugenic writing for professional journals was accidental. A lady patient about four years ago urged him as a duty to present some of the thoughts which had benefited her to the doctors whom she had experienced as lamentably ignorant or careless. In response to her importunity the doctor sent a paper to the TEXAS MEDICAL JOURNAL and others, and within two years he found himself on the contributing list of thirty-five journals, and his work is so much sought after that much of it is syndicated; that is, it is published simultaneously in different journals in different parts of the country. The Doctor is 54 years young, and declares that he is growing younger every day. He is Eugenics Editor of *Utah Medical Journal* and *Denver Medical Times*.

